# Sex Differences in Social Media Addiction: The Mediating Effects of Social Media Use Motives

**DOI:** 10.31083/AP46105

**Published:** 2026-02-25

**Authors:** Young-Jin Lim

**Affiliations:** ^1^Department of Psychology, Gachon University, 13120 Seongnam, Republic of Korea

**Keywords:** internet addiction disorder, sex characteristics, social networking, motivation

## Abstract

**Background:**

The aim of this study was to examine whether social media use motives mediate sex differences in social media addiction.

**Methods:**

Three hundred adults (50.0% women; mean age = 39.28 years, standard deviation = 10.91) in South Korea completed the Social Network Site Use Motives Scale–Revised and the Bergen Social Media Addiction Scale.

**Results:**

Sex differences were found in social media addiction; that is, women reported higher levels of social media addiction than men. In addition, coping motives partially explained the sex differences in social media addiction. Although indirect effects were also observed for enhancement and pastime motives, the effects were not statistically significant after correction for multiple comparisons.

**Conclusion:**

The findings indicate that women are more vulnerable to social media addiction than men, due in part to a difference in coping motives. Thus, interventions targeting coping motives may effectively reduce the risk of social media addiction among Korean adult women.

## Main Points

• Sex predicts information, enhancement, coping, and pastime social media use motives.

• Enhancement, coping, and pastime motives predict social media addiction.

• Coping motives function as a mediator in the relationship between sex and social media addiction.

## 1. Introduction

Social media refers to a virtual space in which individuals express their personal characteristics and share their interests, information, and ideas with others [1]. As of July 2025, about 5.41 billion people, who are approximately 65.7% of the world’s population, used social media [2]. An individual user accessed approximately 6.83 social media platforms per month as of January 2025 [3], and the average daily time spent on social media was 2 hours and 23 minutes as of April 2025 [4].

Social media provides many benefits to social media users [5]. Social media provides access to information, instant communication, educational resources, identity formation through self-expression, social integration, collective participation, and marketing opportunities for disseminating content and services.

Despite its advantages, social media use has various negative outcomes [5, 6, 7]. Excessive social media use causes disrupted circadian rhythms, diminished concentration, poor sleep hygiene, and reduced learning capacity. Social media users may experience feelings of inferiority and low self-esteem because of social comparison. Misinformation, including fake news, pseudoscientific health claims, and conspiracy theories, is spread by social media. Furthermore, certain users utilize social media for harmful purposes, such as cyberbullying, digital harassment, and cyberstalking. Lastly, phubbing, which refers to using social media during face-to-face interactions, can lower the intimacy of interpersonal relationships.

Social media addiction is one of the various negative outcomes of social media use. Social media addiction, which is characterized by excessive engagement with social media and a diminished ability to regulate one’s social media use, is considered a form of behavioral addiction [6]. Individuals with social media addiction report a strong craving to use social media, excessive time spent on social media, weakened interpersonal relationships, and impaired academic or occupational performance.

Social media addiction has six components of behavioral addiction: salience, tolerance, mood modification, withdrawal, relapse, and conflict [6]. Individuals with social media addiction experience salience, in which thoughts and cravings related to social media dominate their thinking and motivations. They use social media to alleviate negative emotions (mood modification); however, when they refrain from using social media, they experience various negative physical and psychological symptoms (withdrawal). Furthermore, the psychological effects of social media tend to diminish over time (tolerance). Individuals with social media addiction often fail to meet social obligations because of their social media use (conflict) and repeatedly fail to reduce or control their use despite efforts to do so (relapse). A meta-analysis on social media addiction that used a tool with these six items and included 35,520 university students found that the global prevalence rate was 18.4%, whereas the rate in Asia was as high as 22.8% [7].

Research has consistently demonstrated that girls and women exhibit higher levels of social media addiction than boys and men [8]. In contrast to these consistent and robust results, the mechanisms underlying women’s greater vulnerability to social media addiction remain unknown. The compensatory Internet use theory (which suggests that individuals use social media to satisfy needs that cannot be met offline) [9], the social comparison theory (which assumes that individuals evaluate themselves based on comparisons with others) [10], and the gender role theory (which emphasizes the influence of social expectations on behavior) [11] have been proposed to explain the sex differences in social media addiction. However, no study has directly investigated whether these differences can be attributed to sex-specific motives for social media use.

**Fig. 1.**
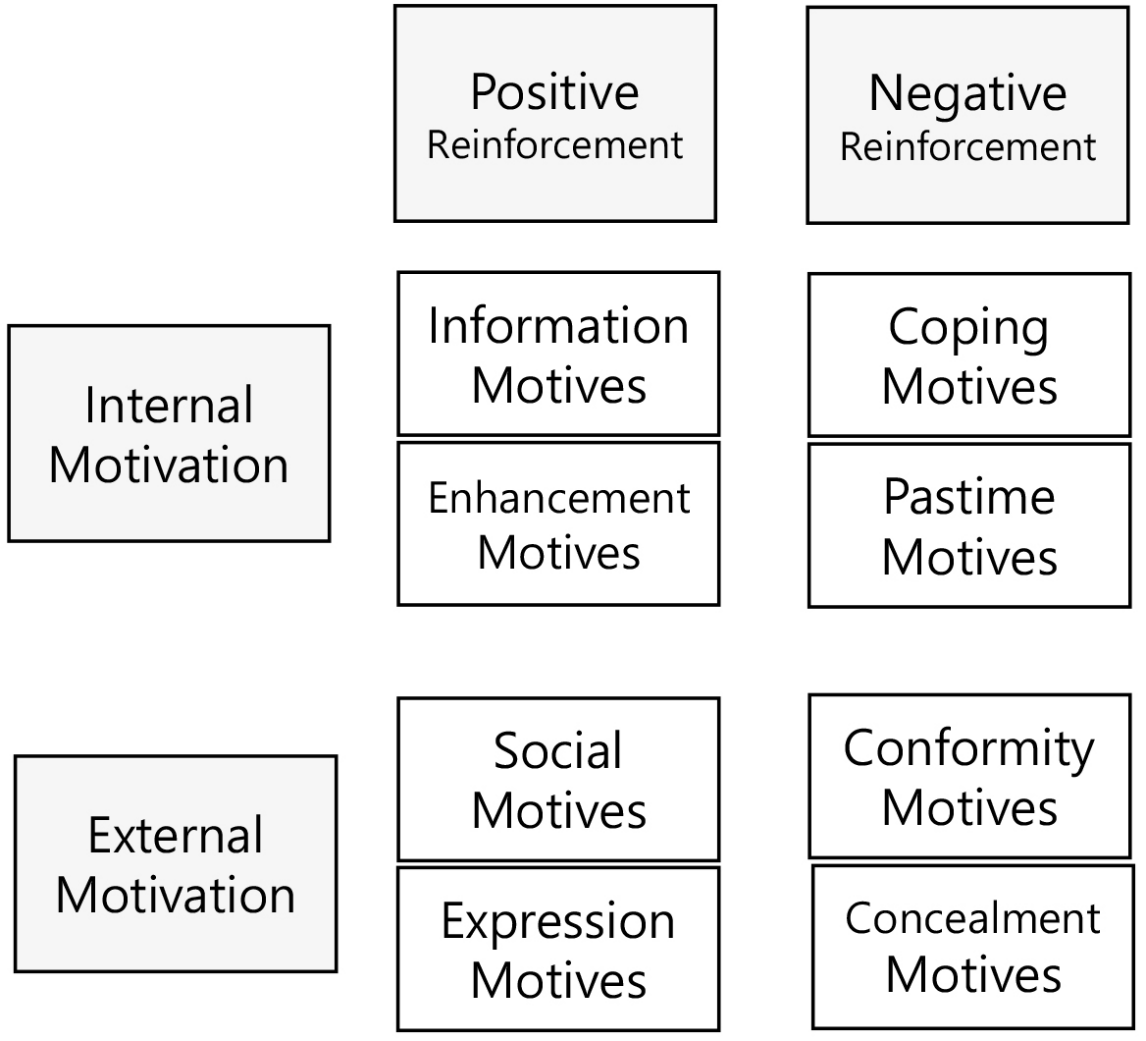
**Social media use motives**.

Social media use motives refer to the reasons individuals use social media based on their experiences with it [6]. A recent model of social media use motives [12] indicated that social media use motives consist of eight specific motives: information, social, enhancement, conformity, coping, pastime, expression, and concealment (Fig. 1).

These eight social media use motives are categorized according to the origin of motivation (internal vs. external) and the nature of reinforcement (positive vs. negative). Internally driven motives associated with positive reinforcement include enhancement and information motives. These two motives reflect a desire for emotional gratification or cognitive stimulation. Internally driven motives associated with negative reinforcement encompass coping and pastime motives, which involve the use of social media to alleviate negative emotional states, such as stress or boredom. Externally driven motives associated with positive reinforcement include social and expression motives, which are related to forming connections and attaining social approval. Finally, externally driven motives associated with negative reinforcement include conformity and concealment motives, which are associated with avoiding social rejection and masking perceived shortcomings.

Social media use motives are related to social media addiction. A previous study found correlation coefficients with small effect sizes between information motives and social media addiction; however, correlation coefficients with moderate effect sizes were observed for enhancement, social, pastime, and expression motives and social media addiction [12]. Correlation coefficients with large effect sizes were found for coping, conformity, and concealment motives and social media addiction. The results of a multiple regression analysis in which eight use motives were entered as predictor variables and social media addiction was input as a criterion variable showed that enhancement, coping, conformity, expression, and concealment motives were associated with social media addiction [13, 14].

Sex differences in social media use motives have been reported, but they have not yet been fully explored. Among the eight social media use motives, women reported higher levels than men for information, enhancement, coping, and pastime motives [13, 14]. Several explanations have been proposed for these results. One explanation is that women experience more negative emotions and have fewer adaptive emotion regulation strategies than men [15, 16]. According to the compensatory Internet use theory and the uses and gratifications theory [17], individuals who experience emotional distress may use social media to alleviate or compensate for negative emotions. From this perspective, women are more inclined to use social media to obtain emotional comfort or to divert their attention away from stressors. They also tend to use social media to enhance their positive emotions while sharing their personal experiences with others [18]. Such patterns are congruent with the self-determination theory, which suggests that people pursue relatedness and the enhancement of positive emotions through social interactions [19].

An empirical study has indicated that social media use motives can serve as mediating variables between various risk factors and social media addiction. For instance, entertainment, communication, and self-expression motives mediate the association between the dark personality traits and social media addiction [20]. Accordingly, social media use motives may serve as a motivational pathway from sex to social media addiction.

**Fig. 2.**
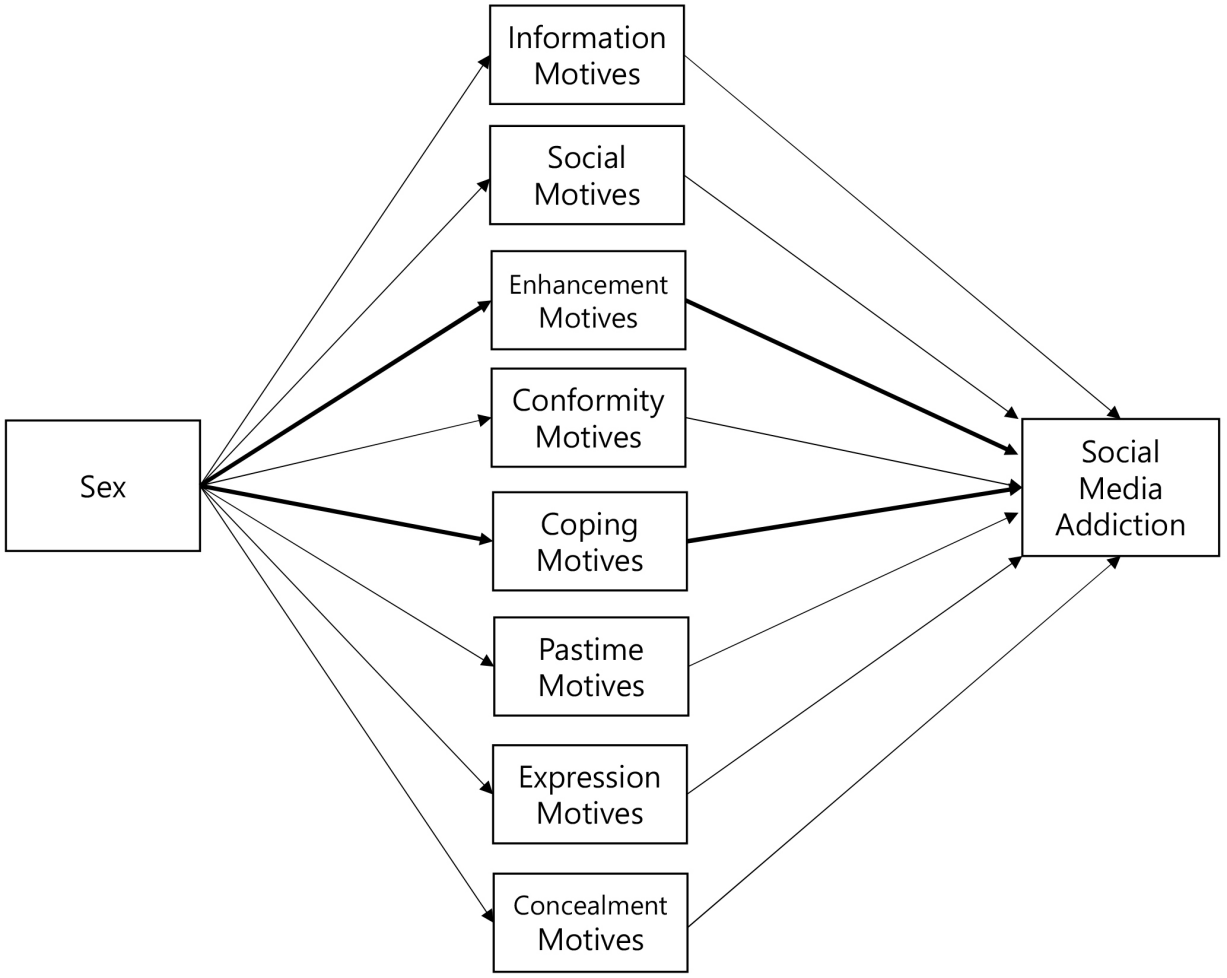
**Conceptual model of the study**. Significant indirect pathways are indicated in bold.

According to previous research, the aim of this study was to examine sex differences in social media addiction and to determine whether social media use motives mediate this association. Fig. 2 presents the proposed hypotheses: (1) women demonstrate significantly greater tendencies toward social media addiction than men, (2) enhancement motives significantly mediate the link between sex and social media addiction, and (3) coping motives significantly mediate the relationship between sex and social media addiction.

This study is the first to examine the mediating effects of social media use motives on the relationship between sex and social media addiction. Previous studies have either focused on sex differences in social media addiction, the relationship between sex and social media use motives [13, 14], or the relationship between social media use motives and social media addiction [12, 13, 14]. Thus, this study offers crucial insights into the relationships between sex, social media use motives, and social media addiction.

## 2. Methods

### 2.1 Participants

The participants were 300 adults proportionally sampled from various regions of Korea. All participants were social media users, and 50% were women. Mean participant age was 39.28 years (standard deviation = 10.91), with an age range of 20–59 years. An a priori power analysis was conducted using G*Power 3.1 (α = 0.05, 1-β = 0.80; Heinrich Heine University Düsseldorf, Düsseldorf, Germany) to determine the necessary sample size when one independent variable, eight parallel mediators, and one covariate were included in the model. The results indicated that approximately 167 participants would be required to detect a medium effect size (*f*^2^ = 0.05) [21]. Tests of indirect effects generally require larger samples [22]; therefore, this study set a target sample size of *N* ≥280 and successfully obtained *N* = 300. Data were collected through Embrain (https://www.embrain.com), an online survey research company. Before beginning the survey, the participants were informed about the purpose of the study, provided informed consent, and received redeemable points as compensation for their participation. The participants in the present study were drawn from the sample used in a previous study [23]. However, this study used different variables, applied a different analytical method, and examined independent hypotheses.

### 2.2 Measures

#### 2.2.1 The Social Network Site Use Motives Scale (SUMS-R)

The SUMS-R is a 40-item self-report measure developed to assess eight distinct social media use motives: information, social, enhancement, conformity, coping, pastime, expression, and concealment [12]. It is a revised version of the original 30-item SUMS. The revised version adds expression and concealment motives to the original version. Each of the eight subscales, which reflect eight use motives, consists of five items each. Respondents indicate the extent of their agreement with each item on a 5-point Likert scale (1 = strongly disagree to 5 = strongly agree). Each subscale score ranges from 5 to 25. Higher scores indicate higher levels of the corresponding motives. The SUMS-R was developed according to the uses and gratifications theory, which asserts that individuals actively choose media to fulfill specific psychological and social needs. In this study, the eight subscales showed adequate internal consistency, with Cronbach’s alpha coefficients that ranged from 0.858 to 0.936.

#### 2.2.2 The Bergen Social Media Addiction Scale (BSMAS)

The BSMAS is a six-item self-report measure designed to assess the level of social media addiction. The BSMAS for adolescents and adults has been demonstrated to have good psychometric properties [24]. Each item of the BSMAS corresponds to one of the six components of behavioral addiction: salience, mood modification, withdrawal, conflict, tolerance, and relapse. Respondents rate each item on a 5-point Likert scale that ranges from 1 (very rarely) to 5 (very often). The total score of the BSMAS ranges from 6 to 30. Higher scores indicate a higher level of social media addiction. Although a universally accepted cutoff score has not been established, scores exceeding 19 have been proposed to indicate social media addiction. In this study, the BSMAS showed adequate internal consistency (Cronbach’s alpha = 0.893).

### 2.3 Data Analyses

Data analysis was conducted using IBM SPSS Statistics (version 27; IBM Corp., Armonk, NY, USA) and Mplus (version 8; Muthén & Muthén, Los Angeles, CA, USA). Prior to the main analysis, Harman’s single-factor test was carried out to assess the presence of common method bias. Descriptive statistics were computed to summarize the demographic characteristics of the sample, Pearson’s correlation coefficients were calculated to examine the associations among the eight social media use motives and social media addiction, and point-biserial correlation coefficients, which can measure the relationship between the variable in the same way as Pearson’s correlation coefficients, were calculated to examine the associations among sex (coded as a binary variable), the eight social media use motives, and social media addiction. A previous study has shown that point-biserial correlation coefficients can show consistent results with Pearson’s correlation coefficients [25]. A parallel multiple mediation analysis was performed to simultaneously examine multiple mediators and assess the unique and independent mediating effects of each variable on the relationship between the independent variable (sex) and the dependent variable (social media addiction). This analysis estimated the indirect effects of each of the multiple social media use motives, controlling for other use motives. In this model, age was a covariate to account for possible confounding effects. Indirect effects were regarded as statistically significant when bias-corrected 95% confidence intervals, which were calculated through bootstrapping with 5000 resamples, excluded zero. The intercorrelations among mediating variables were freely estimated to minimize potential multicollinearity among the eight social media use motives. In addition, all variables were standardized before analysis. This standardization allowed the indirect and direct effects to be interpreted in standard deviation units, reducing multicollinearity and improving the interpretability and comparability of the findings. Effect sizes for the total, direct, and indirect effects were estimated using the κ^2^ (kappa-squared) index proposed by Preacher and Kelley [26]. According to their criteria, κ^2^ values of 0.01, 0.09, and 0.25 represent small, medium, and large effects, respectively. The κ^2^ value indicates the proportion of the maximum possible indirect effect relative to the outcome variance and functions as a standardized index of effect size that aids in interpreting the practical significance of mediation effects.

In this study, the false discovery rate (FDR) correction was applied to all indirect effects to control for potential Type I error inflation caused by multiple comparisons in the parallel multiple mediation model [27]. Model fit was evaluated using the comparative fit index (CFI), Tucker–Lewis index (TLI), root mean square error of approximation (RMSEA), and standardized root mean square residual (SRMR). CFI and TLI values ≥0.95 and RMSEA and SRMR values ≤0.06 and 0.08, respectively, indicated a satisfactory model fit [28].

## 3. Results

Harman’s single-factor test was conducted to examine the potential presence of common method bias. An exploratory factor analysis was conducted on all 46 items used in this study. The results indicated that, among the seven factors with eigenvalues greater than 1, the first factor accounted for 17.38% of the total variance. No single factor explained more than 50% of the total variance; therefore, the likelihood of substantial common method bias was considered low [29].

### 3.1 Descriptive Analysis

Tables 1A and 1B present the descriptive statistics (means, standard deviations, skewness, and kurtosis) and correlation coefficients for the study variables. The results of internal consistency analyses indicated that all scales met the conventional criteria, with reliability coefficients (Cronbach’s alphas) exceeding 0.85, indicating high levels of reliability. The skewness (–0.736 to 0.645) and kurtosis (–0.770 to 0.621) values for all variables fell within the commonly accepted thresholds (|skewness| <3, |kurtosis| <2) [30], suggesting that the assumption of normality was satisfied for each variable. Sex (coded as men = 1 and women = 0) was significantly and negatively associated with four of the eight social media use motives (*r* = –0.122 to –0.159) and social media addiction (*r* = –0.217, *p* < 0.001). Social media addiction was significantly and positively correlated with all social media use motives (*r* = 0.417 to 0.660).

**Table 1A.** **Descriptive statistics for the variables**.

	Mean	SE	SD	Range	Skewness	Kurtosis	Cronbach’s alpha
Information motives	17.04	0.229	3.973	5–25	–0.376	0.298	0.868
Social motives	13.83	0.248	4.298	5–25	0.028	–0.551	0.858
Enhancement motives	16.96	0.229	3.958	5–25	–0.233	0.349	0.896
Conformity motives	11.35	0.248	4.290	5–24	0.244	–0.694	0.899
Coping motives	12.42	0.270	4.679	5–25	0.431	–0.421	0.897
Pastime motives	17.54	0.252	4.372	5–25	–0.736	0.621	0.910
Expression motives	13.01	0.258	4.461	5–25	0.077	–0.607	0.911
Concealment motives	10.52	0.252	4.372	5–24	0.645	–0.062	0.936
Social media addiction	14.70	0.326	5.645	6–30	0.332	–0.770	0.893

Note: n = 300. SE, standard error; SD, standard deviation.

**Table 1B.** **Correlation coefficients**.

	1	2	3	4	5	6	7	8	9	10
1. Sex	-									
2. Information motives	–0.15*	-								
3. Social motives	–0.02	0.32***	-							
4. Enhancement motives	–0.12*	0.68***	0.47***	-						
5. Conformity motives	–0.09	0.25***	0.76***	0.33***	-					
6. Coping motives	–0.16**	0.42***	0.53***	0.52***	0.62***	-				
7. Pastime motives	–0.13*	0.44***	0.26***	0.65***	0.20***	0.51***	-			
8. Expression motives	–0.09	0.43***	0.67***	0.45***	0.53***	0.52***	0.31***	-		
9. Concealment motives	–0.05	0.30***	0.52***	0.29***	0.59***	0.61***	0.26***	0.62***	-	
10. Social media addiction	–0.22***	0.42***	0.48***	0.54***	0.49***	0.66***	0.49***	0.49***	0.44***	-

Note: n = 300; *p < 0.05, **p < 0.01, ***p < 0.001.

### 3.2 Parallel Multiple Mediation Analysis

The parallel multiple mediation analysis requires several statistical assumptions to be satisfied. In this study, the assumptions of homoscedasticity and linearity were not violated, and no univariate or multivariate outliers were detected. Tables 2,3,4 present the results of the parallel multiple mediation analysis examining the relationships between sex, social media use motives, and social media addiction.

**Table 2.** **Parameter estimates from the parallel multiple mediation model**.

Path		β	SE	t	*p* value	95% confidence interval	
						LLCI	ULCI
Sex → Various Social Media Use Motives							
	Sex → Information Motives	–0.147	0.056	–2.643	0.008	–0.258	–0.035
	Sex → Social Motives	–0.019	0.057	–0.334	0.738	–0.132	0.090
	Sex → Enhancement Motives	–0.123	0.056	–2.211	0.027	–0.229	–0.013
	Sex → Conformity Motives	–0.087	0.057	–1.531	0.126	–0.200	0.021
	Sex → Coping Motives	–0.159	0.057	–2.814	0.005	–0.267	–0.048
	Sex → Pastime Motives	–0.134	0.055	–2.424	0.015	–0.243	–0.026
	Sex → Expression Motives	–0.089	0.057	–1.561	0.119	–0.202	0.020
	Sex → Concealment Motives	–0.047	0.058	–0.803	0.422	–0.164	0.065
Various Social Media Use Motives → Social Media Addiction (SMA)							
	Sex → SMA	–0.104	0.043	–2.425	0.015	–0.196	–0.026
	Information Motives → SMA	0.005	0.056	0.082	0.935	–0.108	0.110
	Social Motives → SMA	0.024	0.068	0.353	0.724	–0.107	0.161
	Enhancement Motives → SMA	0.170	0.070	2.427	0.015	0.039	0.313
	Conformity Motives → SMA	0.110	0.078	1.403	0.161	–0.039	0.240
	Coping Motives → SMA	0.365	0.078	4.660	<0.001	0.203	0.509
	Pastime Motives → SMA	0.111	0.047	2.344	0.019	0.017	0.203
	Expression Motives → SMA	0.100	0.070	1.435	0.151	–0.032	0.241
	Concealment Motives → SMA	–0.007	0.066	–0.107	0.915	–0.145	0.113

Note: SMA, social media addiction; LLCI, lower limit of confidence interval; ULCI, upper limit of confidence interval.

**Table 3.** **Total and direct effects of sex on social media addiction**.

	Estimate	SE	Est./SE	*p* value	95% confidence interval	
					LLCI	ULCI
Total effect	–0.217	0.055	–3.985	<0.001	–0.326	–0.108
Direct effect	–0.104	0.043	–2.425	0.015	–0.196	–0.026

Note: Est, estimate.

**Table 4.** **Indirect effects of sex on social media addiction**.

	Estimate	SE	κ ^2^	95% confidence interval		FDR-corrected *p*-value
				LLCI	ULCI	
Total effect	–0.113	0.041	0.298	–0.195	–0.035	-
Information	–0.001	0.009	0.000	–0.019	0.017	>0.05 *(ns)*
Social	0.000	0.004	0.000	–0.016	0.005	>0.05 *(ns)*
Enhancement	–0.021	0.013	0.010	–0.056	–0.003	0.078
Conformity	–0.010	0.010	0.002	–0.043	0.002	>0.05 *(ns)*
Coping	–0.058	0.026	0.079	–0.119	–0.016	0.013
Pastime	–0.015	0.009	0.005	–0.040	–0.002	0.074
Expression	–0.009	0.009	0.002	–0.040	0.001	>0.05 *(ns)*
Concealment	0.000	0.005	0.000	–0.008	0.014	>0.05 *(ns)*

Note: κ^2^, effect size (0.010 = small; 0.090 = medium; 0.250 = large); FDR, false discovery rate; *ns*, not significant.

The model was just-identified (df = 0); therefore, global model fit indices were not interpreted.

As Table 2 shows, the direct effects of sex on information (β = –0.147, *p* = 0.008), enhancement (β = –0.123, *p* = 0.027), coping (β = –0.159, *p* = 0.005), and pastime (β = –0.134, *p* = 0.015) motives were statistically significant. Furthermore, the direct effects of the mediators on social media addiction were significant for enhancement (β = 0.170, *p* = 0.015), coping (β = 0.365, *p* < 0.001), and pastime (β = 0.111, *p* = 0.019) motives.

Table 3 shows that the total effect of sex on social media addiction was statistically significant (β = –0.217, *p* < 0.001), as was the direct effect (β = –0.104, *p* = 0.015). Table 4 shows that the total indirect effect of sex on social media addiction was also significant (point estimate = –0.113, 95% CI [–0.195, –0.035]), with a small-to-medium effect size. Indirect effects via enhancement (point estimate = –0.021, 95% CI [–0.056, –0.003]), coping (point estimate = –0.058, 95% CI [–0.119, –0.016]), and pastime (point estimate = –0.015, 95% CI [–0.040, –0.002]) motives were statistically significant. In addition, FDR correction was applied to all indirect effects. The coping motive (*p* FDR = 0.013) remained statistically significant after the correction, whereas the adjusted *p*-values for enhancement and pastime motives were 0.078 and 0.074, respectively, exceeding the 0.05 significance threshold. Therefore, enhancement and pastime motives were considered relatively less significant mediators. These findings support Hypothesis 3, indicating that coping motives mediate the relationship between sex and social media addiction.

## 4. Discussion

Although prior research has identified significant associations between sex and social media addiction, the motivational mechanisms underlying this relationship remain unexplored. Consistent with previous findings [8], this study found higher levels of social media addiction in women. Furthermore, significant sex differences were observed in four of the eight social media use motives. All social media use motives were significantly and positively associated with social media addiction, which is consistent with the previous findings [13, 14].

In addition, this study examined whether enhancement and coping motives could mediate the relationship between sex and social media addiction. The analysis showed that coping motives were the only significant mediating variable, whereas other motives, including enhancement motives, were not significant mediators. These results demonstrate that sex is not related to social media addiction equally through all use motives but through specific motives.

The bootstrapping analysis showed that the 95% CI for the total indirect effect was statistically significant, the proportion of the total indirect effect was higher than the proportion of the direct effect, and the κ^2^ effect size for the total indirect effect was large, indicating practical significance. The κ^2^ effect size for the indirect effect of coping motives was in the small-to-medium range, demonstrating that sex meaningfully predicted social media addiction via coping motives.

Consistent with the hypothesis, coping motives significantly mediated the relationship between sex and social media addiction. These findings suggest that coping motives serve as the primary pathway linking sex to social media addiction. The finding that coping motives significantly mediated the relationship between sex and social media addiction can be explained by two factors. First, in Korea, there is a strong social expectation that women should regulate and suppress their emotions in their daily lives, which may lead them to release negative emotions in social media spaces [31]. Second, in general, Korean women tend to experience more mental health problems, such as depression and anxiety, than men [32, 33], and because negative perceptions and social stigma of psychological difficulties and mental health problems still exist, they may try to use social media as a coping mechanism to relieve psychological pain.

The mediating role of coping motives can be explained through the compensatory Internet use theory [9] and gender role theory [11]. According to the compensatory Internet use theory, individuals use online platforms to compensate for negative emotions experienced in offline contexts. From this perspective, coping motives can be understood as compensatory strategies to alleviate psychological distress through social media use. Moreover, the gender role theory suggests that women in collectivistic cultures, such as South Korea, are socialized to suppress emotional expression and maintain relational harmony. Accordingly, women may rely more heavily on social media as a socially acceptable means of emotional regulation.

The finding that enhancement motives did not significantly mediate the relationship between sex and social media addiction did not support the hypothesis. The indirect effect was not significant, despite the paths from sex to enhancement motives and from enhancement motives to social media addiction all being significant. We can explain this finding in two ways. First, indirect effects can be calculated as the product of two paths. Even when both paths are statistically significant, their product may not be sufficiently large to produce a statistically significant indirect effect, particularly if the resulting effect size is small relative to the sampling error. Second, the statistical power to detect mediation effects can be influenced by sample size. Considering the relatively small sample in this study, the power to detect modest indirect effects might have been insufficient.

Methodological issues aside, there are two possible explanations for why the enhancement motives did not function as a mediating variable in this study. First, sensation seeking, a potential antecedent of the enhancement motives, is more commonly observed in adolescents and males, so its effect may have been weakened in the general adult sample. Second, Korean adults’ social media use tends to be motivated more by social connectedness than by the enhancement of positive emotions, which may have weakened the mediating role of the enhancement motives.

The current study showed that both the direct effect of sex on social media addiction and the indirect effect through social media use motives were statistically significant. This finding suggests that social media use motives did not fully explain the relationship between sex and social media addiction. Future research that identifies and incorporates additional use motives would improve the explanatory capacity of the model. For example, coping motives could be divided into sub-motives for coping with depression, anxiety, and anger. Thus, revision of the existing eight-motive model and/or scale is warranted.

The findings have theoretical implications. The results of this study enhanced understanding of the relationship between sex and social media addiction by integrating the literature on sex differences and theories on social media use motives. Furthermore, the findings have practical implications for preventing social media addiction. Prevention programs that target coping motives may be particularly effective for women, according to the findings from this study. For example, women who use social media for coping with negative emotions may reduce their vulnerability to social media addiction by developing alternative emotion regulation strategies. Furthermore, for women who have negative attitudes toward mental health and are consequently hesitant to express feelings of depression or anxiety openly, interventions aimed at modifying such negative perceptions may be particularly effective.

This study has some limitations that should be pointed out. First, this study employed a cross-sectional design, so causal inferences cannot be drawn. Future studies should replicate or confirm the findings of the current study using a longitudinal design. Second, although Harman’s single-factor test showed a low likelihood of common method bias, depending solely on self-report questionnaires still has several potential limitations. Future research should replicate these findings using diverse measurement methods, such as behavioral or observational methods. Third, this study did not include different types of social media platforms as variables. Previous studies have shown that social media platforms vary in terms of usage time, intensity, and user motives [1, 6]. Future research should consider platform-specific characteristics when examining social media use. Fourth, the scale used in this study to measure social media use motives includes eight factors. However, these eight motives may not reflect the full spectrum of social media use motives. Thus, further revisions and refinements of the scale are needed. Finally, comparative analysis among different regions within South Korea with varying levels of competitive social pressure was not conducted in the study; thus, it is difficult to determine whether such competitive social pressure, which is characteristic of Korean culture, contributes to higher level of social media addiction among Korean women than among Korean men.

## 5. Conclusion

This study demonstrated the presence of motivational mechanisms linking sex and social media addiction. However, future research including longitudinal designs, utilizing diverse measurement tools, applying revised scales that assess social media use motives, and incorporating information on specific social media platforms is needed. Such advanced research could provide a more comprehensive and nuanced understanding of the relationship between sex and social media addiction. Despite the limitations, the findings of the present study can contribute to the development and design of prevention programs for women at risk of social media addiction.

## Availability of Data and Materials

The data and materials supporting the findings of this study are available from the corresponding author upon reasonable request, in accordance with the journal’s editorial policies on data availability.
